# Therapeutic Strategies for the Management of Hormone Receptor-Positive, Human Epidermal Growth Factor Receptor 2-Positive (HR+/HER2+) Breast Cancer: A Review of the Current Literature

**DOI:** 10.3390/cancers12113317

**Published:** 2020-11-10

**Authors:** Eirini Thanopoulou, Leila Khader, Morena Caira, Andrew Wardley, Johannes Ettl, Federica Miglietta, Patrick Neven, Valentina Guarneri

**Affiliations:** 1 Eli Lilly and Company Limited, Erl Wood Manor, Windlesham, Surrey GU20 6PH, UK; eirini.thanopoulou@roche.com; 2 Eli Lilly Italia S.p.A., 50019 Comune di Sesto Fiorentino, Florence, Italy; khader_leila@lilly.com (L.K.); morenacaira@yahoo.it (M.C.); 3 The NIHR Manchester Clinical Research Facility at The Christie NHS Foundation Trust, School of Medical Sciences, Faculty of Biology Medicine & Health, University of Manchester, Manchester M204BX, UK; Andrew.Wardley@manchester.ac.uk; 4 Department of Obstetrics and Gynecology, Klinikum rechts der Isar, Technical University of Munich, 81675 Munich, Germany; johannes.ettl@tum.de; 5 Medical Oncology 2, Istituto Oncologico Veneto IOV IRCCS, 35128 Padua, Italy; federica.miglietta@iov.veneto.it; 6 Department of Surgery, Oncology and Gastroenterology, University of Padova, 35128 Padua, Italy; 7 Multidisciplinary Breast Center and Department of Gynecology and Obstetrics, UZ Leuven, 3000 Leuven, Belgium; patrick.neven@uzleuven.be; 8 Department of Oncology, KU Leuven, 3000 Leuven, Belgium

**Keywords:** advanced breast cancer, early breast cancer, cyclin-dependent kinase 4/6 inhibitors, hormone receptor-positive, human epidermal growth factor receptor 2-positive

## Abstract

**Simple Summary:**

In the last decades, tremendous advances have been made in understanding HER2-positive breast cancer biology, with a progressive improvement in survival rates of patients with this breast cancer subtype. However, a not negligible proportion of patient with HER2-positive breast cancer will eventually relapse, and metastatic HER2-positive disease is still to be considered an incurable condition, thus highlighting the imperative need to further improve our understanding in this regard. In this context, there is growing knowledge that HER2-overexpressing breast tumors are highly heterogeneous, and the co-expression of hormone-receptors may account, at least in part, for this heterogeneity. The aim of the present work is to review preclinical and clinical evidence on HER2-positive/hormone-receptor positive breast cancer, focusing on studies investigating both activity and efficacy of various combination of treatment strategies, including anti-HER2 drugs, hormonal treatments and other targeted agents, such as CDK inhibitors, both in the early and advanced setting.

**Abstract:**

Enormous advances have been made in the understanding and treatment of human epidermal growth factor receptor 2-positive breast cancer (HER2+ BC) in the last 30 years that have resulted in survival gains for affected patients. A growing body of evidence suggests that hormone receptor-positive (HR+)/HER2+ BC and HR-negative (HR−)/HER2+ BC are biologically different, with complex molecular bidirectional crosstalk between the estrogen receptor and HER2 pathway potentially affecting sensitivity to both HER2-targeted and endocrine therapy in patients with HR+/HER2+ BC. Subgroup analyses from trials enrolling patients with HER2+ BC and the results of clinical trials specifically designed to evaluate therapy in patients with HR+/HER2+ BC are helping to guide treatment decisions. In this context, encouraging results with strategies aimed at delaying or reversing drug resistance, including extended adjuvant therapy and the addition of drugs targeting alternative pathways, such as cyclin-dependent kinase (CDK) 4 and 6 inhibitors, have recently emerged. We have reached the point where tailoring the treatment according to risk and biology has become the paradigm in early BC. However, further clinical trials are needed that integrate translational research principles and identify and consider specific patient subgroups and biomarkers.

## 1. Introduction

The identification of human epidermal growth factor receptor 2 (HER2) as a prognostic and predictive factor and target for therapy both defined HER2-positive breast cancer (HER2+ BC) and revolutionized treatment for this aggressive type of BC, improving patient survival in early BC (EBC) and advanced BC (ABC) [1,2]. The development of new drugs targeting HER2 has progressively improved patient outcomes to the extent that HER2+ BC is, in many cases, no longer such a devastating type of BC [3]. However, the substantial heterogeneity of HER2+ disease means a large group of patients require new therapeutic strategies. The majority of patients with HER2+ metastatic BC (MBC) still die of BC after many years of continuous treatment. The simultaneous expression of hormone receptors (HR+) accounts for one easily identifiable aspect of this heterogeneity, and HR+/HER2+ disease affects approximately 10% of patients with BC [4,5]. The behavior of HR+/HER2+ BC frequently differs from that of HR−/HER2+ BC, and the two subtypes are increasingly being recognized as requiring different therapeutic approaches. HR+/HER2+ BC is a distinct subtype that presents clinical challenges in view of treatment optimization [6,7,8,9,10,11].

The co-expression of HRs in HER2+ BC is consistently associated with more favorable clinicopathologic features than HR−/HER2+ BC, such as a lower stage based on the American Joint Committee on Cancer at diagnosis, lower histologic grade, and—in the case of triple-positive BC (estrogen receptor (ER+), progesterone receptor (PgR+), and HER2+ BC)—smaller median tumor size [12]. Patients with ER+/HER2+ BC also tend to have a more linear pattern of mortality than those with ER−/HER2+ BC, with relapse often occurring later than that in patients with ER−/HER2+ BC, who tend to relapse earlier [13,14].

Genomic heterogeneity, identified using next-generation sequencing (NGS), segregates HER2+ BC into at least two major clinically distinct entities: luminal BC and HER2-enriched (HER2E) BC, which have profoundly different gene expression, mostly related to ER signaling [15]. This genomic heterogeneity has substantial implications with respect to the sensitivity to HER2-targeted treatment [16]. Primary medical therapy with either single [16] or dual HER2-targeted therapy [17] is much more likely to result in a pathologic complete response (pCR) in HER2E BC than in luminal HER2+ BC.

Treatment guidelines for EBC recommend endocrine (neo) adjuvant therapy for all patients with HR+ BC and HER2-targeted therapy for patients with HER2+ BC. When both HR and HER2 are present, complexity and apparent conflict may arise. The recommended treatment strategy for patients with HR+/HER2+ BC is chemotherapy plus HER2-targeted therapy (trastuzumab with or without pertuzumab), followed by endocrine therapy [6,11]. Similarly, the current guidelines for ABC specify that all suitable patients with HER2+ BC should be offered chemotherapy plus HER2-targeted therapy with trastuzumab plus pertuzumab; when chemotherapy is stopped, maintenance endocrine therapy plus HER2-targeted therapy is suggested [7,11]. Selected patients with HR+/HER2+ MBC can be offered HER2-targeted therapy in combination with endocrine therapy as the initial therapy; these patients include those with contraindications to, or a strong preference not to receive, chemotherapy and those with a long disease-free interval, minimal disease burden, and/or strong ER/PgR expression [7].

Novel treatment strategies, often targeting diverse signaling pathways, have been proposed to further improve the outcomes in this clinical setting. The current article comprehensively reviews preclinical and clinical data involving HER2+ BC, focusing on the HR+/HER2+ subpopulation, and current treatment strategies that have been validated preclinically and tested in clinical trials in various settings. Noteworthy, in this context, combinations of targeted agents, including the association of the HER2 blockade with endocrine therapy, as well as CDK 4 and 6 inhibitors, show some promise, since CDK 4 and 6 activity are typically dysregulated and overactive in BC [18]. The use of CDK 4 and 6 inhibitors warrants exploration in this setting, particularly when the aim is to de-escalate chemotherapy.

## 2. Factors Affecting the Response and Resistance to HER2- and HR-Targeted Therapies

**Figure 1:**
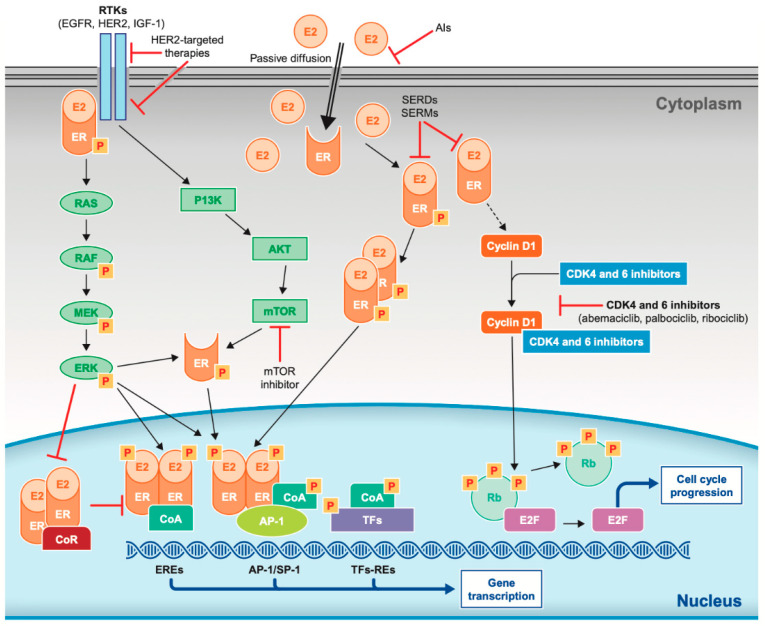
Receptor pathways involved in the progression of breast cancer and mechanism of action of endocrine and targeted therapies. AI = aromatase inhibitor; AKT = protein kinase B; CDK = cyclin-dependent kinase; CoA = coactivator complex; CoR = corepressor complex; E2 = estradiol; EGFR = epidermal growth factor receptor; ER = estrogen receptor; EREs = estrogen receptor elements; ERK = extracellular signal-regulated kinase; HER2 = human epidermal growth factor receptor 2; IGF-1 = insulin-like growth factor 1 receptor; P = phosphorylation; PI3K = phosphatidylinositol 3-kinase; mTOR = mammalian target of rapamycin; Rb = Retinoblastoma protein (tumor suppressor protein); RE = response elements; RTKs = receptor tyrosine kinases; SERD = selective estrogen receptor degrader; SERM = selective estrogen receptor modulator; and TFs = transcription factors (e.g., activator protein 1 (AP-1), specificity protein 1 (SP-1), and E2 factor (E2F).

Receptor mechanisms involved in the progression of BC are summarized in Figure 1.

### 2.1. HER2 Itself

The major determinant of the HER2-targeted treatment response is HER2 itself. “Oncogene addiction” is used to describe tumors fully dependent on HER2 for proliferation and survival. These cancers are often exquisitely sensitive to HER2-directed therapy [16,17], even in the absence of chemotherapy [19]. The presence of HER2 in the putative cancer stem cell may play an important role in the achievement of a favorable outcome for patients with this type of BC.

Intrinsic or acquired resistance to HER2-targeted treatment can occur when HER2 mutations are present or cleavage of the extracellular domain of HER2 by matrix metalloproteases occurs [19].

Conversely, the hyperactivity of HER2 (or the HER2 downstream signaling pathway) may interfere with ER expression and activity, thus representing a potential escape pathway in HR+ BC overexpressing HER2 and determining a state of de novo endocrine resistance [20].

### 2.2. Hormone Receptors

HER2+ BC that is HR+ (ER+ and/or PgR+) is emerging as a biologically and clinically distinct entity enriched for the luminal gene clusters (e.g., *GATA3*, *BCL2*, and *ESR1*) [15,19,21]. HER2 enrichment positively, and luminal subtype (ER+ expression) negatively, predicts the response to trastuzumab plus paclitaxel-based therapy [22].

Complex molecular bidirectional crosstalk between the ER and HER2 pathways may perpetuate tumor growth and survival [19,23,24,25], with signaling through ER being a preferred escape pathway to HER2 inhibition [23,26,27,28] and HER2 being a preferred escape from ER inhibition [29,30,31]. ER may enhance HER2 signaling activity at both the genomic and the nongenomic level by, respectively, promoting the expression of ligands of diverse growth factor receptors (including those belonging to the HER family) and directly interacting with HER2, triggering the downstream cascade [23,32,33]. Conversely, HER2 may mediate post-translational modifications, causing both enhanced ER genomic activity and reduced estrogen dependency via the HER1-mitogen-activated protein kinase pathway [30,34,35,36,37]. Preclinical data suggest that ER signaling is involved in de novo and acquired resistance to HER2 pharmacological inhibition. Both upstream ER regulators and downstream ER effectors, as well as ER itself, may be upregulated in response to a lapatinib-mediated HER2 blockade, thus, ultimately, resulting in cell growth and proliferation restoration [23]. The concurrent inhibition of ER and HER2 can improve outcomes. Hence, targeting pathways implicated in this crosstalk appears to be a possible strategy to delay or reverse drug resistance [23].

### 2.3. PI3K/AKT/mTOR Pathway

The dysregulation of signaling downstream from HER2+ may lead to escape from HER2-targeted and HR-targeted therapy [23]. Constitutive activation of the phosphatidylinositol 3-kinase/protein kinase B/mammalian target of rapamycin (PI3K/AKT/mTOR) pathway, via mutations of the PI3K catalytic subunit (PIK3CA; encoding for the p110α protein) or complete/partial loss of the phosphatase and tensin homolog (PTEN; a suppressor of the PI3K pathway), is important [38] and is a potential target for new therapies.

Activating mutations of the *PIK3CA* gene, seen in about 20% to 30% of HER2+ tumors and 30% to 35% of HR+ BCs [19,39,40], are associated with worse outcomes in clinical trials of HER2-targeted therapies than wild-type *PIK3CA* [19,40,41,42,43], particularly if the BC is also HR+ [42,43]. Conversely, BC with high HER2 amplification and an intact PI3K pathway appears to be especially sensitive to HER2-targeted neoadjuvant therapy in the absence of chemotherapy [43,44]. Mutations in a large network of *PIK3CA*-related genes may confer a differential resistance to specific HER2-targeted therapies [45].

### 2.4. Immune-Related

A growing body of evidence suggests that immune components of the tumor microenvironment may affect sensitivity to systemic treatments and prognosis. High levels of tumor-infiltrating lymphocytes (TILs) are associated with higher pCR rates after preoperative chemotherapy plus HER2-targeted neoadjuvant treatment [46,47,48] and improved outcomes (overall survival (OS)) with chemotherapy plus HER2-targeted therapy in patients with ABC [49]. In addition, despite conflicting evidence with regard to the prognostic/predictive role of TILs in the neoadjuvant setting [46,47,48,50,51,52], NGS analysis suggested a potential predictive role for immune-gene enrichment in terms of the response to the HER2 blockade [44,46,47,53]. The relationship between HR and the immune system is complex, encompassing inflammation and its players, immune cells, estrogens, and the immunomodulatory action of endocrine and HER2-targeted therapy [19,44,48,50,51,52]. Preclinical evidence suggests that endocrine therapy is capable of preventing the estrogen-mediated shift of tumor-associated macrophages from M1 to the more protumorigenic M2. Preclinical and clinical evidence suggests that aromatase inhibitors (AIs) dampen T-cell regulatory activity, promote cytotoxic T-cell activity, and foster a proinflammatory status in the context of the tumor microenvironment [51]. Research into the use of CDK4 and 6 inhibitors for the management of advanced HR+ BC has fostered a better understanding of the possible interactions between CDK4 and 6 and the immune system. Preclinical studies suggest that CDK4 and 6 might enhance T-cell activity through several mechanisms involving attenuation of activity of the nuclear factor of activated T cells-family proteins and their target genes and the transcriptional repression of early growth response 1 [54,55]. Notably, the pharmacological inhibition of CDK4 and 6 was reported to increase in vivo T-cell activation and TIL levels [56].

## 3. Clinical Trials in HR+/HER2+ BC

**Figure 2:**
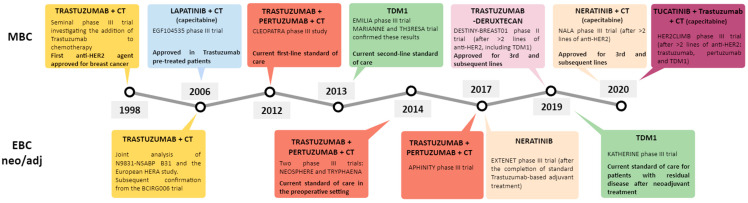
The evolution of human epidermal growth factor receptor 2 (HER2) blockade strategies in HER2+ breast cancer. CT = chemotherapy, EBC = early breast cancer, HER2 = human epidermal growth factor receptor 2, MBC = metastatic breast cancer, neo/adj = (neo) adjuvant, and TDM1 = trastuzumab emtansine.

HER2-targeted treatment has dramatically changed outcomes for patients with HER2+ BC, improving survival in both EBC and ABC (Figure 2). Outcomes for some patients with HER2+ EBC are now so improved that a focus for current research is to maintain efficacy using less intensive regimens with fewer toxicities and better tolerability. This is the focus of a new generation of clinical trials in which patients with low-to-moderate-stage HER2+ EBC have achieved a pCR to treatment. Patients without a pCR or with ABC require additional therapeutic strategies.

### 3.1. Metastatic Setting

Phase II and III trials investigating treatment strategies for patients with HR+/HER2+ MBC are summarized in Table 1.

Dual HER2-targeted therapy regimens, utilizing trastuzumab and pertuzumab plus a taxane, are the preferred standard of care for the first-line management of HER2+ MBC, irrespective of HR status [7,11], following the phase III CLEOPATRA trial showing improved progression-free survival (PFS) and OS with these regimens when compared with trastuzumab plus docetaxel therapy in patients with HER2+ ABC [41,60]. The end-of-study results of this trial revealed that patients treated with the pertuzumab-containing regimen had an OS of 57.1 months (95% confidence interval (CI) 50–72), compared with 40.8 months (95% CI 36–48) for those treated with the placebo-containing regimen, and that the eight-year OS rates were 37% (95% CI 31–42) and 23% (19–28), respectively [41]. Endocrine therapy was excluded in this trial, yet is frequently added after the completion of taxane therapy in routine practice, as endorsed in the guidelines [7] and supported by the registHER and SystHER observational studies, which showed clinically meaningful improvements in outcomes of patients with HR+/HER2+ BC treated with maintenance endocrine therapy [75,76].

The phase II PERNETTA trial showed a two-year median OS to be similar when trastuzumab and pertuzumab were administered with or without chemotherapy as the first-line therapy (75.0 vs. 74.2 months in HR+/HER2+ BC) [64]. Irrespective of HR status, the chemotherapy-free regimen resulted in a significantly shortened progression-free survival (PFS), but such regimens may be of value for patients who are unable or unwilling to receive chemotherapy.

In the second- and subsequent-line management of HR+/HER2+ MBC, trastuzumab emtansine (T-DM1) provided a survival advantage in patients who progressed on the previous treatment with trastuzumab plus chemotherapy [67,68,69]. In the recently reported HER2CLIMB trial, the addition of tucatinib to trastuzumab and capecitabine resulted in a clinically significant and meaningful improvement in PFS and OS compared with trastuzumab and capecitabine therapy (OS median 21.9 vs. 17.4 months; hazard ratio 0.66; 95% CI 0.50–0.88; *p* = 0.005) in patients with HER2+ MBC previously treated with trastuzumab, pertuzumab, and T-DM1. There was consistent benefit across subgroups, including patients with HR+ disease and, importantly, in patients with brain metastases [70].

In the phase II DESTINY-Breast01 study, the treatment with trastuzumab deruxtecan (DS-8201) showed high response rates (overall response rate (ORR) 60.9%) and durable antitumor activity (median PFS 16.4 months and median response duration 14.8 months) in patients with HER2+ MBC who underwent extensive previous treatment. Prespecified subgroup analyses showed consistent responses across demographic and prognostic subgroups, including patients with HR+ status who had an ORR of 58% (56 of 97 patients) [69].

Both tucatinib and trastuzumab deruxtecan are now licensed in several countries and are likely to be important additions to the treatment pathway where funded.

#### 3.1.1. Combination ER/HER2-Targeted Therapy

Targeting HER2 and HR simultaneously was investigated as a strategy for patients with HR+/HER2+ BC, with encouraging ORR and PFS, suggesting that chemotherapy-free options for the palliative management of patients with HR+/HER2+ BC are a reasonable option. The phase II TAnDEM study randomized HR+/HER2+ postmenopausal patients (*n* = 207) to receive either anastrozole plus trastuzumab or anastrozole alone [57]. Previous endocrine therapy in EBC was permitted, whereas chemotherapy and a HER2-targeted treatment for either EBC or ABC was not. Compared with single-agent anastrozole, the combination therapy resulted in improved PFS (4.8 vs. 2.4 months; hazard ratio 0.63; 95% CI 0.47‒0.84; *p* = 0.0016) and a nonsignificant trend toward improved OS (28.5 vs. 23.9 months), in spite of a 70% crossover to a trastuzumab-containing regimen on progression in the anastrozole arm.

In EGF30008, which randomized patients with HR+ BC (*n* = 1171) to receive letrozole plus either lapatinib or placebo, the predetermined HR+/HER2+ subgroup (*n* = 219) had improved PFS (8.2 vs. 3.0 months; hazard ratio 0.71; 95% CI 0.53‒0.96; *p* = 0.019) and clinical benefits rate (CBR; stable or responding disease ≥6 months: 48% vs. 29%; odds ratio (OR) 0.4; 95% CI 0.2‒0.8; *p* = 0.003) with dual therapy [59]. Although immature (less than 50% of OS events recorded), the analysis of available data failed to show a statistically significant OS improvement with the combination arm (median OS 32.3 months for letrozole vs. 33.3 months for letrozole plus lapatinib; hazard ratio 0.74; 95% CI 0.5–1.1; *p* = 0.113).

The phase III eLEcTRA trial compared the combination of letrozole plus trastuzumab with letrozole alone as a first-line therapy in postmenopausal women with HR+/HER2+ MBC (*n* = 57) [58]. Median PFS was 14.1 months in patients randomized to the combination compared with 3.3 months in those receiving letrozole alone. However, the study was relatively small, and statistical significance was not achieved (hazard ratio 0.67; *p* = 0.23). The respective CBRs were 65% and 39% (OR 2.99; 95% CI 1.01‒8.84).

In the ALTERNATIVE trial, postmenopausal women with HR+/HER2+ MBC (*n* = 355) were randomly assigned to lapatinib plus trastuzumab, lapatinib, or trastuzumab (all with an AI and without chemotherapy) [62]. All patients had received prior trastuzumab and endocrine therapy in the early (76%) and/or metastatic settings (30%). PFS was significantly increased by lapatinib plus trastuzumab plus AI, as compared with trastuzumab plus AI (11 vs. 5.7 months; hazard ratio 0.62; 95% CI 0.45‒0.88; *p* = 0.0064). ORR was also numerically higher with the dual HER2-targeted combination plus AI compared with trastuzumab plus AI (31.7% vs. 13.7%). Similarly, results of the phase II PERTAIN study, in which postmenopausal women (*n* = 258) were randomly assigned to first-line pertuzumab plus trastuzumab and an AI or trastuzumab plus an AI, showed an improved PFS with the three-drug combination (18.9 vs. 15.8 months; hazard ratio 0.65; 95% CI 0.48‒0.89; *p* = 0.0070) [63]. This PFS benefit was also maintained when considering only patients who did not receive induction chemotherapy (hazard ratio 0.55; 95% CI 0.34–0.88; median PFS 21.72 vs. 12.45 months; *p* = 0.011).

The combination therapies were associated with a higher incidence of toxicities, especially diarrhea and rash, than single-agent HER2-targeted or endocrine therapy [57,59,63], which needs to be weighed against any benefits with respect to efficacy.

Together, the results of trials investigating the combination of HER2-targeted therapy plus endocrine therapy suggest that, for selected patients with HR+/HER2+ ABC, such strategies may be effective for those wishing to avoid chemotherapy.

#### 3.1.2. HER2-Targeted Therapy Plus Additional Targeted Therapy

Several trials have investigated the combination of HER2-based treatment with different targeted agents in patients with HER2+ ABC, with variable results seen in the HR+/HER2+ subpopulation.

The phase III BOLERO-1 trial evaluated the addition of the mTOR inhibitor everolimus to trastuzumab plus paclitaxel as a first-line treatment, and the phase III BOLERO-3 trial evaluated the addition of everolimus to trastuzumab plus vinorelbine for trastuzumab-resistant patients with HER2+ BC [66,72]. In both trials, a modest PFS benefit observed with the addition of everolimus was at the cost of increased toxicity. Interestingly, everolimus appeared to improve PFS more in the populations with HR− BC, with no PFS difference observed in the HR+ subgroups. A biomarker analysis revealed that patients with HER2+ ABC and *PIK3CA* mutations, PTEN loss, or a hyperactive PI3K pathway derived the greatest PFS benefit from everolimus [77].

Ongoing phase I trials evaluating the addition of PI3K inhibitors to HER2 blockade plus chemotherapy have conflicting preliminary results. The combination of trastuzumab plus the PI3Ka inhibitor alpelisib and the HER3 inhibitor LJM716 in patients with heavily pretreated *PIK3CA*-mutated HER2+ MBC had limited activity, possibly as a result of the substantial associated toxicity (diarrhea, mucositis, hyperglycemia, increased liver enzymes, and hypokalemia) [78]. In contrast, alpelisib, in combination with T-DM1 in unselected heavily pretreated patients with HER2+ MBC (47% of which was HR+), produced encouraging ORR (43%), including an ORR of 30% in the ten T-DM1-resistant patients, and PFS rates (8.1 months; 95% CI 3.9–10.8) [79]. Buparlisib (a pan-class I PI3K inhibitor) plus lapatinib showed preliminary evidence of antitumor activity, with a manageable safety profile, in heavily pretreated patients with HER2+ trastuzumab-resistant MBC, 50% of whom had HR+ disease [80].

The addition of CDK4 and 6 inhibitors to treatment regimens, including HER2-targeted therapies in heavily pretreated patients, appears promising (Table 1). The PATRICIA study randomized 30 postmenopausal patients with HR+/HER2+ MBC, who had been previously treated with two to four lines of anti-HER2-based therapy, to receive palbociclib plus trastuzumab with or without letrozole [73]. CBRs were 40% and 53.3% in the respective treatment groups, which meant the criteria of PFS at six months of 40% were met, and stage 1 of the study was successful. A phase I study evaluating abemaciclib, as monotherapy or with continued endocrine therapy, in 47 women with ABC who had received a median of seven (range 2‒16) prior systemic therapies showed partial responses in 4/11 patients (36%) with HR+/HER2+ ABC [81]. Collectively, these results encouraged the clinical development of CDK4 and 6 inhibitors in combination with HER2-targeted therapies for patients with HR+/HER2+ BC.

The phase II monarcHER trial randomized 237 patients with HR+/HER2+ ABC to receive abemaciclib plus trastuzumab plus fulvestrant vs. abemaciclib plus trastuzumab vs. trastuzumab plus the investigator’s choice of chemotherapy [74]. The majority of patients had visceral disease. Almost all patients had received prior endocrine and HER2-targeted treatments. An efficacy analysis revealed a statistically significant improvement in PFS (8.3 vs. 5.7 months; hazard ratio 0.67) and ORR (confirmed ORR 33% vs. 14%) with the combination of abemaciclib plus trastuzumab plus fulvestrant as compared with trastuzumab plus chemotherapy. No PFS or ORR difference was observed between abemaciclib plus trastuzumab and trastuzumab plus chemotherapy. No new safety signals were identified beyond those already reported in BC trials. A triplet combination of a CDK4 and 6 inhibitor, endocrine therapy, and HER2-targeted therapy may be an alternative active and effective treatment option in heavily pretreated patients with HR+/HER2+ ABC. Further evidence is anticipated from ongoing clinical trials testing CDK4 and 6 inhibitors, endocrine agents, and HER2-targeted agents in both the first and subsequent lines of treatment in HR+/HER2+ ABC or MBC.

### 3.2. Neoadjuvant Setting

Phase II and III trials investigating neoadjuvant treatment strategies for patients with HR+/HER2+ EBC are summarized in Table 2. The accelerated approval of pertuzumab as a primary medical therapy has seen a paradigm shift in the treatment of HER2+ EBC, with neoadjuvant trastuzumab and pertuzumab plus chemotherapy the preferred option for patients in whom HER2-targeted therapy is indicated [6,11]. Trastuzumab and lapatinib plus chemotherapy has also demonstrated some efficacy in this setting [82].

Results from the clinical trials showed that a dual HER2-targeted blockade (trastuzumab plus lapatinib or pertuzumab) in combination with neoadjuvant chemotherapy for HER2+ BC greatly increased pCR compared with a single HER2-targeted blockade (lapatinib or trastuzumab) plus chemotherapy (Table 2) [44,82,83,85,86,87]. However, the co-expression of HER2 and HR may have major implications for the treatment response. The findings of these studies showed that pCR rates are generally lower in the subpopulation with HR+/HER2+ BC as compared with that with HR−/HER2+, irrespective of the treatment (Table 2). Promising results with dual HER2-targeted blockade without chemotherapy in patients with HR−/HER2+ BC (pCR rate of 27.3%) in the NeoSphere trial were not seen in patients with HR+/HER2+ BC (pCR rate of 5.9%) [86]. Taken together, these data support the different biology of HR+/HER2+ BC, which may need different treatment approaches in the future, including neoadjuvant treatment for longer than 12 weeks [90].

#### 3.2.1. Combination ER/HER2-Targeted Therapy

The simultaneous administration of endocrine therapy and HER2-targeted therapy plus chemotherapy-based neoadjuvant treatment has been investigated. The preliminary evidence suggests that the addition of hormonal therapy to HER2-targeted therapy plus chemotherapy-based neoadjuvant treatment may enhance the response in patients with HR+/HER2+ BC (Table 2). The NSABP B52 phase III trial randomized patients with HR+/HER2+ BC (*n* = 315) to receive neoadjuvant chemotherapy plus a dual HER2-targeted blockade (trastuzumab plus pertuzumab) with or without an AI (plus a luteinizing hormone-releasing hormone analog (LHRHa) if premenopausal) [95]. A nonstatistically significant increase in pCR rates was observed with the addition of endocrine therapy to the HER2-targeted therapy plus chemotherapy regimen, with no significant impact on toxicity.

The addition of endocrine therapy to HER2-targeted neoadjuvant treatment without chemotherapy was evaluated in the TBCRC006 study. Patients (*n* = 64) with locally advanced HER2+ BC received a 12-week course of a dual HER2-targeted blockade with trastuzumab and lapatinib; patients with HR+ BC also received letrozole (plus LHRHa if premenopausal). The pCR (ypT0-is) rate was 27% in the overall intention-to-treat population, but the authors reported promising rates of the protocol-specified pathologic response (ypT0-is + ypT1a-b) in patients with HR+/HER2+ BC (13/40; 33%) after a short course of HER2-targeted therapy plus endocrine treatment [28]. In the follow-up TBCRC023 trial, which included 97 patients with HER2+ BC and compared a 12-week vs. a 24-week regimen of trastuzumab plus lapatinib (with letrozole—plus LHRHa if premenopausal—if ER+), the pCR rates after 24 weeks of neoadjuvant treatment were 12.1% vs. 27.9% at 12 vs. 24 weeks in the overall population [90]. The differences in the pCR rates observed between the 12-week and 24-week neoadjuvant treatments were almost entirely driven by the ER+ subpopulation (*n* = 62, 8.7% vs. 33.3% with 12 vs. 24 weeks of treatment), suggesting that a longer exposure to endocrine therapy plus a dual HER2-targeted blockade may be worth exploring further.

In PAMELA, a single-group open-label trial in patients with HER2+ BC, different regimens were administered for HR+ and HR− BC. The pCR rate in patients with HR+/HER2+ BC, who received 18 weeks of trastuzumab plus lapatinib plus letrozole (*n* = 77), was 18%, compared with 43% in those with HR−/HER2+ BC (*n* = 74), who received only trastuzumab plus lapatinib (*p* = 0.0015). An analysis based on the baseline PAM50 results revealed that, among patients with HR+/HER2+ BC, 32% of the HER2E subgroup achieved pCR in the breast, as compared with 5% of patients with non-HER2E BC [89].

In the PerELISA study in 61 evaluable postmenopausal patients with HR+/HER2+ operable BC, following two weeks of letrozole, molecular responders (Ki67 relative reduction >20% from the baseline at two weeks) continued endocrine therapy in combination with dual HER2-targeted blockade with trastuzumab and pertuzumab, whereas molecular nonresponders switched to taxane-based chemotherapy plus dual HER2-targeted blockade. The pCR rate was 20.5% (95% CI 11.1–34.5) among molecular responders and 81.3% in molecular nonresponders. These results suggest that meaningful pCR rates can be achieved in some molecular responders using de-escalated treatment without chemotherapy [91], thus indicating that Ki67 may be a promising tool for the selection of patients who may benefit from a de-escalated chemotherapy-free neoadjuvant treatment when chemotherapy is not an option. Indeed, the proliferation marker Ki67 is considered an important prognostic factor in EBC [6] and can be used for guiding decisions on the adjuvant therapy choice, as well as for predicting the response to neoadjuvant treatment [96,97]. The PerELISA study confirmed the observation from the PAMELA trial reporting higher rates of pCR in a HER2E subtype among molecular responders (45.5% vs. 13.8%; *p* = 0.042) [89,91].

The results of the PHERGain study showed a pCR of 35% among the subgroup of patients with ER+/HER2+ EBC treated with neoadjuvant trastuzumab plus pertuzumab plus endocrine therapy who had a positron emission tomography (PET) response after two cycles of treatment; the PET response was predictive of the pCR [92].

#### 3.2.2. Alternative Regimens

The ADAPT trial, a large (*n* = 375) phase II, open-label, multicenter study specifically conducted in patients with HR+/HER2+ BC, compared 12-week regimens of T-DM1 with or without endocrine therapy vs. trastuzumab plus endocrine therapy [88]. The authors reported that, whereas trastuzumab plus endocrine therapy produced unsatisfactory pCR rates, T-DM1 was associated with remarkable and clinically meaningful pCR rates, with no benefit provided by the addition of endocrine therapy (pCR rates: 15.1% vs. 41.0% vs. 41.5%). The KRISTINE trial showed that, in patients with HER2+ BC, traditional neoadjuvant systemic chemotherapy plus a dual HER2-targeted blockade (docetaxel, carboplatin, trastuzumab, and pertuzumab) produced a significantly higher pCR than T-DM1 plus pertuzumab (55.7% vs. 44.4%; *p* = 0.016) but was associated with numerically more grade 3/4 and serious adverse events than the T-DM1-containing regimen [93].

A number of ongoing clinical trials suggest that a multilevel inhibition of HER2, ER, and CDK4 and 6 blockade may be promising for the neoadjuvant management of triple-positive BC. In the single-arm phase II NA-PHER trial, the combination of trastuzumab–pertuzumab–fulvestrant–palbociclib was feasible, safe, and effective in reducing the Ki67 expression from the baseline to week two and produced encouraging rates of both the complete clinical response (50%) and pCR (27%) after 16 weeks of treatment [94]. Several ongoing trials are currently testing the same hypothesis, but this approach remains investigational.

Overall, although available data provide a strong rationale for adjusting neoadjuvant treatment according to HR status in patients with HER2+ BC, solid evidence on long-term outcomes is currently lacking, and no formal recommendation can be made.

### 3.3. Adjuvant Setting

Trials in the adjuvant setting investigating treatment strategies for patients with HER2+ BC, including HR+ BC, are summarized in Table 3.

The addition of trastuzumab to adjuvant chemotherapy dramatically improved both disease-free survival (DFS) and OS as compared with chemotherapy alone in patients with HER2+ EBC [110], and this regimen is currently approved in patients with HER2+ node-positive BC and in patients with node-negative disease and tumors >1 cm [6]. Although adjuvant trastuzumab administered for one year remains the standard therapeutic approach, a shorter duration of trastuzumab may be considered in selected patients with very low-risk features [6]. Trials have tested whether shorter durations of HER2-targeted treatment could reduce both the cardiac toxicity and financial costs without detrimentally affecting the patient survival. Most did not meet the prespecified noninferiority boundaries [98,99,100,101], with the exception of the PERSEPHONE trial [102] (Table 3). In PERSEPHONE (*n* = 4089), a six-month course of trastuzumab was statistically noninferior to a one-year course, with less cardiotoxicity and fewer severe adverse events. The majority of patients (69%) included in PERSEPHONE had HR+ disease, and a subgroup analysis revealed that, although the HR− population may have derived a greater benefit from one year of HER2-targeted treatment, the two regimens were equivalent in the HR+ population (Table 3). Similarly, even when considering negative trials, subgroup analyses revealed the comparable efficacy of one year vs. a shorter trastuzumab duration in patients with favorable clinicopathologic features, including HR+ status. A pooled analysis of individual patient data from the above-mentioned trials may help strengthen this hypothesis, and a meta-analysis of the PERSEPHONE and PHARE trials is planned.

While the aforementioned trials looked to reduce exposure to HER2-targeted therapy, several trials tested escalated treatment strategies for high-risk patients with HER2+ EBC. In the HERA trial, 5102 women were randomized to receive either trastuzumab for one or two years or no further treatment after the completion of adjuvant chemotherapy with curative intent. Two years of trastuzumab did not improve the DFS as compared with one year of treatment, and this lack of benefit from prolonged HER2-targeted treatment was particularly evident in patients with HR+ disease [103,111]. Overall, asymptomatic or mildly symptomatic cardiac endpoints occurred more frequently in patients who received trastuzumab for two vs. one years.

In the APHINITY trial, a dual HER2-targeted blockade with trastuzumab plus pertuzumab statistically significantly improved the invasive DFS (IDFS)—albeit, by a marginally clinically relevant amount—compared with trastuzumab alone, irrespective of HR status [105]. The result was driven by the node-positive population (hazard ratio for an invasive-disease event 0.77; 95% CI 0.62–0.96; *p* = 0.02). International guidelines currently recommend considering the addition of pertuzumab to trastuzumab and chemotherapy only in high-risk patients—namely, those who are node-positive and have HR− BC—thus limiting the role for this escalated treatment strategy in patients with HR+/HER2+ BC [6]. The results of the KAITLIN study shed further light on these strategies. In this study, 1846 patients with node-positive HR+/HER2+ BC achieved similar three-year IDFS rates with three to four cycles of anthracycline-based chemotherapy, followed by 18 cycles of T-DM1 plus pertuzumab (95.4%) or anthracycline-based chemotherapy, followed by taxane plus concurrent trastuzumab plus pertuzumab (94.1%) [106]. Notably, these rates were similar to those in the total HER2+ population (93.1% vs. 94.2%).

Promising results were achieved with tyrosine kinase inhibitor (TKI) therapy in the adjuvant setting in the phase III ExteNET trial [107,108]. A total of 2840 patients who had completed neoadjuvant and adjuvant chemotherapy plus trastuzumab, with no evidence of disease recurrence or metastatic disease, were randomized to receive the TKI neratinib (*n* = 1420) or placebo (*n* = 1420) for one year as an extended adjuvant therapy. The significant improvement in the IDFS rate with neratinib was driven by improvements in patients with HR+ BC (hazard ratio 0.60; 95% CI 0.43–0.83 for HR+ BC vs. 0.95 and 95% CI 0.66–1.35 for HR− BC), most of whom also received standard adjuvant endocrine therapy [108].

The presence of residual disease after neoadjuvant chemotherapy is associated with an adverse outcome. In the phase III KATHERINE trial, patients with invasive residual disease after trastuzumab plus chemotherapy-based neoadjuvant treatment were randomized to either continue trastuzumab for one year after surgery or switch to T-DM1 [109]. T-DM1 significantly increased IDFS compared with trastuzumab (hazard ratio 0.50; 95% CI 0.39–0.64; *p* < 0.001), establishing this as a new standard of care in this setting. The survival benefit provided by adjuvant T-DM1 was consistent across all subgroups, including those with HR+ BC (three-year IDFS for T-DM1 vs. trastuzumab: 90.1% vs. 80.7%; hazard ratio 0.48; 95% CI 0.35–0.67), and irrespective of the amount of residual disease. Given the absence of convincing data supporting the omission of chemotherapy in the adjuvant setting, the guidelines suggest offering the combination of trastuzumab plus endocrine therapy only when chemotherapy is contraindicated or refused by the patient [6].

In the ALTTO trial, a dual HER2-targeted blockade with trastuzumab plus lapatinib did not significantly increase DFS as compared with trastuzumab alone, regardless of HR status [104], and has not changed in practice.

Treatment strategies for premenopausal women with EBC differ somewhat from those in postmenopausal women with EBC. Tamoxifen for five to 10 years is a standard of care for premenopausal women, with concomitant ovarian function suppression (OFS) also recommended for those requiring ovarian protection or who recover menses, and in high-risk patients [6,11]. The recommendations followed the disclosure of the results of the SOFT trial, which revealed a greater treatment effect with tamoxifen plus OFS than with tamoxifen alone (DFS hazard ratio, 0.78; 95% CI 0.62–0.98; *p* = 0.03) [112]. In this trial, the addition of OFS to tamoxifen appeared particularly effective in patients with HER2+ BC (DFS hazard ratio, 0.41; 95% CI 0.22–0.75) compared with those with HER2− BC (0.83; 95% CI 0.67–1.04) [112]. The combined SOFT and TEXT trial results showed that patients with HER2− BC had a higher eight-year DFS rate if assigned to a treatment with exemestane plus OFS (88.1%) rather than tamoxifen plus OFS (82.7%; hazard ratio 0.70; 95% CI 0.60–0.83) [112]. Notably, among 282 premenopausal Asian women with ER+ BC, those with HER2+ tumors may have received greater benefits, in terms of DFS and OS, from adjuvant oophorectomy and tamoxifen vs. no adjuvant therapy than those with HER2− BC [113].

## 4. Conclusions

Enormous advances have been made in the treatment and understanding of HER2+ BC in the last 30 years, and the addition of new therapies has resulted in survival gains in both EBC and ABC. A growing body of evidence suggests that HR+/HER2+ BC and HR−/HER2+ BC are biologically different. In HR+/HER2+ BC, a complex molecular bidirectional crosstalk between the ER and HER2 pathways may be crucial in affecting sensitivity to both HER2-targeted therapy and endocrine treatment [23,24,26]. Subgroup analyses from trials enrolling patients with HER2+ BC and the results of clinical trials specifically designed to evaluate therapy in patients with HR+/HER2+ EBC and ABC are helping to guide treatment decisions. However, careful trial design and the consideration of heterogeneity between BCs is important. To date, drug development has generally targeted one or other of the two best known drivers of BC growth and survival: ER and HER2. It is now well-known that there are complex interactions between ER and HER2, as well as other known therapeutic targets.

Although improved outcomes have been observed with the use of dual HER2-targeted regimens compared with a single HER2 blockade in patients with HER2+ BC, including those with HR+ tumors, in the advanced setting, almost all patients ultimately experience disease progression, and additional treatment strategies are needed to overcome resistance [27]. Some of the recent strategies aimed toward delaying or reversing drug resistance include extended adjuvant therapy in EBC and the addition of targeted agents, such as CDK4 and 6 inhibitors, in ABC. Indeed, there is a strong rationale to evaluate CDK4 and 6 inhibitors in HR+/HER2+ BC, since CDK4 and 6 pathways have been reported to be involved in HER2-targeted therapy resistance, and the pharmacological inhibition of CDK4 and 6 was shown to restore cancer cell sensitivity to the HER2 blockade [18,27,114]. In this context, CDK4 and 6 inhibitors may represent an appealing strategy for chemotherapy de-escalation, as a reduction in toxicity is an important objective, especially in the palliative setting of advanced disease. Moreover, chemotherapy-free treatment options may facilitate care for some patients [19] and may be a particularly compelling approach among vulnerable patient populations, including the elderly and those with poor performance status [18], as well as for patients who refuse chemotherapy.

The identification of additional diagnostic and prognostic biomarkers for targeted therapies is important to improve the applicability and effectiveness of the treatment while reducing the toxicity [18], and further work is needed in this area. Future clinical trials must integrate translational research principles, identifying and considering specific subgroups and biomarkers to help enrich the study population and guide the trial design.

In conclusion, HR+/HER2+ BC is a distinct molecular subtype, and both HR and HER2 pathways are implicated in carcinogenesis and the development of resistance. The strategy of combining a HER2 blockade with hormonal therapy and/or CDK4 and 6 inhibitors may provide an opportunity to address the need to de-escalate chemotherapy in patients with HR+/HER2+ BC. Although much progress has been made in improving the clinical outcomes, further clinical trials are needed that are specifically designed to evaluate new treatment strategies in patients with HR+/HER2+ BC.

## Figures and Tables

**Table 1:** Phase II and III trials in the metastatic setting evaluating the outcomes in patients with HR+/HER2+ breast cancer.

Study Acronym (Study Population)	No. of pts	Treatment Regimen	Results for PFS (PE) in pts with HR+/HER2+ ABC		Comment
			PFS (Months)	Hazard Ratio (95% CI) *p*-Value	
**First-line therapy**					
*ET ± single HER2-targeted therapy*					
Trastuzumab					
TAnDEM [57] All pts HR+/HER2+	103	Trastuzumab + anastrozole	PFS: 4.8 OS: 28.5 2-year OS: 57%	PFS: 0.63 (0.47‒0.84) *p* = 0.0016	OS did not differ significantly between arms, but 70% of the anastrozole arm crossed over to a trastuzumab-containing regimen on progression Trastuzumab + anastrozole improved PFS, TTP, and ORR compared with anastrozole alone but increased (serious) adverse events
	104	Anastrozole	PFS: 2.4 OS: 23.9 2-year OS: 50%		
eLEcTRA [58] All pts HR+/HER2+	26	Trastuzumab + letrozole	TTP: 14.1 2-year PFS: 40%	TTP: 0.67 (0.35–1.29) *p* = 0.23	OS did not differ significantly between arms Trastuzumab + letrozole was safe and effective
	31	Letrozole	TTP: 3.3 2-year PFS: 25%		
Lapatinib					
EGF30008 [59] Subgroup HR+/HER2+ (overall HR+)	111	Lapatinib + letrozole	PFS: 8.2 OS: 33.3	PFS: 0.71 (0.53‒0.96) *p* = 0.019	The ITT population included pts with HR+/HER2− BC and HR+/HER+ BC Lapatinib + letrozole significantly enhanced PFS and clinical benefit rates compared with letrozole in pts with HR+/HER2+ BC OS data were immature at the time of the analysis
	108	Letrozole	PFS: 3.0 OS: 32.3		
*Dual vs. single HER2-targeted therapy*					
CLEOPATRA [41,60] Subgroup HR+/HER2+ (overall HER2+)	189	A: Trastuzumab + pertuzumab + docetaxel *	NR	PFS: 0.72 (0.55–0.95) OS: 0.74 (0.58–0.96)	Hazard ratio PFS for ER−/HER2+ BC: 0.55 (0.42–0.72) Hazard ratio OS for ER−/HER2+ BC: 0.64 (0.50–0.81) In the overall population, PFS was 18.5 months (A) vs. 12.4 months (B); hazard ratio PFS: 0.62 (0.51–0.75; *p* < 0.001). OS was 57.1 (A) vs. 40.8 months (B), hazard ratio OS: 0.69 (0.58–0.82; *p* < 0.001), indicating benefit for addition of pertuzumab to trastuzumab + docetaxel
	199	B: Trastuzumab + placebo + docetaxel *	NR		
MARIANNE [61] Subgroup HR+/HER2+ (overall HER2+)	198	A: T-DM1 + pertuzumab	NR	NR	PFS for ER−/HER2+ BC: (B) 13.3 months, (C): 14.0 months; hazard ratio PFS (B vs. C) for ER−/HER2+ BC: 1.00 (0.73–1.37) In the overall population, median PFS was 15.2 (A), 14.1 (B), and 13.7 months (C); B was noninferior to C (hazard ratio PFS: 0.91; 97.5% CI 0.73–1.13; *p* = 0.31); the addition of pertuzumab to T-DM1 did not improve PFS
	195	B: T-DM1 + placebo	PFS: 13.4	PFS: B vs. C: 0.94 (97.5% CI 0.71–1.25)	
	207	C: Trastuzumab + taxane	PFS: 13.7		
ALTERNATIVE [62] All pts HR+/HER2+ **	120	A: Lapatinib + trastuzumab + AI	PFS: 11.0 OS: 46.0	PFS: A vs. B (comparison for PE): 0.62 (0.45‒0.88) *p* = 0.0064 C vs. B: 0.71 (0.51–0.98) *p* = 0.0361 OS: A vs. B: 0.60 (0.35–1.04) C vs. B: 0.82 (0.49–1.36)	PFS benefit for dual HER2-targeted therapy vs. trastuzumab was seen in pts receiving prior trastuzumab in the (neo)adjuvant (hazard ratio PFS: 0.70 (0.47–1.05)) or metastatic setting (hazard ratio PFS: 0.44 (0.23–0.83)) OS data were immature at the time of analysis but trended in favor of dual HER2-targeted therapy
	117	B: Trastuzumab + AI	PFS: 5.7 OS: 40.0		
	118	C: Lapatinib + AI	PFS: 8.3 OS: 45.1		
PERTAIN [63] All pts HR+/HER2+	129	Pertuzumab + trastuzumab + AI ± taxane at clinician discretion	PFS: 18.9	PFS: 0.65 (0.48‒0.89) *p* = 0.0070	The PFS benefit of dual HER2-targeted therapy was seen in pts who received induction taxane therapy (hazard ratio PFS: 0.75 (0.50–1.13)) and in those who did not (hazard ratio PFS: 0.55 (0.34–0.88))
	129	Trastuzumab + AI ± taxane at clinician discretion	PFS: 15.8		
*Timing of chemotherapy*					
PERNETTA [64] Subgroup HR+/HER2+ (overall HER2+)	NR	A: Trastuzumab + pertuzumab, then T-DM1 at progression	PFS: 8.3 (90% CI 6.3–13.5) 2-year OS: 75.0% (90% CI 64.9–83.4)	NR	PFS for ER−/HER2+ BC: (A) 8.8 months (90% CI 7.9–14.6), (B) 22.2 months (90% CI 11.4–32.6) 2-year OS for ER−/HER2+ BC: (A) 81.1% months (90% CI 67.4–90.8), (B) 79.5% months (90% CI 66.0–89.4) HR status did not affect first-line PFS In the overall population (N = 210), first-line PFS was shorter with A (8.4 months (7.7–12.0) vs. B: 23.3 months (17.6–32.6)), but 2-year OS was the same in both arms (A: 76.2% (68.4–82.9) vs. B: 76.2% (68.4–82.9))
	NR	B: Trastuzumab + pertuzumab + paclitaxel or vinorelbine	PFS: 23.7 (90% CI 18.2–33.8) 2-year OS: 74.2% (90% CI 63.9–82.9)		
*HER2-targeted therapy plus additional targeted therapy*					
Anti-VEGF agent					
AVEREL [65] Subgroup HR+/HER2+ (overall HER2+)	115	Bevacizumab + trastuzumab + docetaxel	NR	PFS: 0.81 (0.59–1.11)	Hazard ratio PFS for ER−/HER2+ BC: 0.81 (0.59–1.12) In the overall population, hazard ratio PFS: 0.82 (0.65–1.02); *p* = 0.078, so no significant advantage for addition of bevacizumab in the total HER2+ population or HR status subpopulations
	107	Trastuzumab + docetaxel	NR		
mTOR inhibitor					
BOLERO-1 [66] Subgroup HR+/HER2+ (overall HER2+)	271	A: Everolimus + trastuzumab + paclitaxel	NR	NR	PFS for ER−/HER2+ BC: (A) 20.3 months (15.0–24.1), (B) 13.1 months (10.1–16.6); hazard ratio PFS for ER−/HER2+ BC: 0.66 (0.48–0.91); *p* = 0·0049 (not significant) In the overall population, treatment A did not improve PFS: 15 months (14.5–17.9) vs. B: 14.5 months (12.3–17.1); hazard ratio 0.89 (0.73–1.08); *p* = 0.117)
	135	B: Placebo + trastuzumab + paclitaxel	NR		
**Second-line or later therapy**					
EMILIA [67] (subgroup HR+/HER2+) (overall HER2+) ***	282	A: T-DM1	NR	PFS: 0.72 (0.58–0.91)	Hazard ratio PFS for ER−/HER2+ BC: 0.56 (0.44–0.72) In the overall population, PFS: (A) 9.6 months, (B) 6.4 months; hazard ratio PFS: 0.65 (0.55–0.77); *p* < 0.001 2-year OS for A vs. B: 64.7% (59.3–70.2) vs. 51.8% (45.9–57.7)
	263	B: Lapatinib + capecitabine	NR		
TH3RESA [68,69] (subgroup HR+/HER2+) (overall HER2+) ^†^	208	A: Physician’s choice (trastuzumab-containing regimen: 80%, lapatinib + chemotherapy: 3%, single-agent chemotherapy: 17%)	PFS: 3.9 OS: 16.4	PFS: 0.56 (0.41–0.76) OS: 0.71 (0.52–0.97)	PFS for ER−/HER2+ BC: (A) 2.9 months, (B) 6.0 months; hazard ratio PFS for ER−/HER2+ BC: 0.51 (0.37–0.71) OS for ER−/HER2+ BC: (A) 15.5 months, (B) 21.2 months; hazard ratio OS for ER−/HER2+ BC: 0.65 (0.46–0.90) In the overall population, PFS: (A) 6.2 months (5.6–6.9), (B) 3.3 months (2.9–4.1); hazard ratio PFS: 0.53 (0.42–0.66); *p* < 0.0001 In the overall population, OS: (A) 15.8 months (13.9–18.7), (B) 22.7 months (19.4–27.5); hazard ratio OS: 0.69 (0.55–0.86); *p* = 0.0007
	103	B: T-DM1	PFS: 5.9 OS: 26.3		
HER2CLIMB [70] Subgroup HR+/HER2+ (overall HER2+) ^‡^	190 (PE) 243	A: Trastuzumab plus capecitabine plus tucatinib	NR	PFS: 0.58 (0.42–0.80) OS: 0.85 (0.59–1.23)	Hazard ratio PFS for ER−/HER2+ BC: 0.54 (0.34–0.86) Hazard ratio OS for ER−/HER2+ BC: 0.50 (0.31–0.80) In the PE population, PFS: (A) 7.8 months (7.5–9.6), (B) 5.6 months (4.2–7.1); hazard ratio PFS: 0.54 (0.42–0.71); *p* < 0.001 In the overall population, 2-year OS: (A) 44.9% (36.6–52.8), (B) 26.6% (15.7–38.7); hazard ratio PFS: 0.66 (0.50–0.88); *p* = 0.005
	99 (PE) 127	B: Trastuzumab plus capecitabine plus placebo	NR		
DESTINY-Breast01 [71] Subgroup HR+/HER2+ (overall HER2+) ^††^	97	Trastuzumab deruxtecan	ORR (PE): 58% (47–68)		ORR for ER−/HER2+ BC: 66% (55–76) In the overall population, ORR: 60.9% (53.4–68.0) In the overall population, PFS: 16.4 months (12.7–not reached)
*HER2-targeted therapy plus additional targeted therapy*					
mTOR inhibitor					
BOLERO-3 [72] Subgroup ER+/PgR+/HER2+ (overall HER2+) ***	317 (total ER+/PgR+)	A: Everolimus + trastuzumab + vinorelbine	NR	PFS: 0.93 (0.72–1.20)	Hazard ratio PFS for ER−/HER2+ BC: 0.65 (0.48–0.87) In the overall population, PFS: (A) 7.0 months (6.7–8.2), (B) 5.8 months (5.5–6.9) hazard ratio PFS: 0.78 (0.65–0.95); *p* = 0.0067
		B: Placebo + trastuzumab + vinorelbine	NR		
CDK4 and 6 inhibitor					
PATRICIA [73] Subgroup HR+/HER2+ (overall HER2+) ^‡‡^	15	A: Palbociclib + trastuzumab + letrozole	6-month PFS: 40.0%	NR	6-month PFS for ER−/HER2+ BC: C (Palbociclib + trastuzumab): 33.3% PFS did not differ significantly between A (all ER+/HER2+ MBC), B (all ER+/HER2+ MBC), and C (all ER−/HER2+ MBC)
	15	B: Palbociclib + trastuzumab	6-month PFS: 53.3%		
monarcHER [74] All HR+/HER2+ ^†††^	79	A: Abemaciclib + trastuzumab + fulvestrant	PFS: 8.3	A vs. B PFS (PE): 0.67 (0.45–1.00); *p* = 0.051 ^‡‡‡^ C vs. B PFS: 0.94 (0.64–1.38); *p* = 0.77	PFS was significantly prolonged by 2.6 months with a chemotherapy-free regimen of abemaciclib + trastuzumab + fulvestrant (A) compared with standard-of-care chemotherapy + trastuzumab (B)
	79	B: Trastuzumab + clinician’s choice of single-agent chemotherapy	PFS: 5.7		
	79	C: Abemaciclib + trastuzumab	PFS: 5.7		

* Patients could not receive ET in this study. ** Prior treatment with ET and disease progression during or after a trastuzumab plus chemotherapy regimen in the (neo)adjuvant setting and/or in the first-line metastatic setting was required (maximum one prior regimen in the metastatic setting). *** Patients had previously received trastuzumab and a taxane. ^†^ Patients had previously received trastuzumab and lapatinib (advanced setting) and a taxane (any setting). ^‡^ Patients had previously received trastuzumab, pertuzumab, and T-DM1. ^††^ Patients had previously received T-DM1 and trastuzumab; patients had received a median of 6 (2–27) previous lines of therapy, including pertuzumab (66%). ^‡‡^ Patients had received two to four prior lines of HER2-targeted therapy-based regimens. ^†††^ Most patients had received previous endocrine therapy (77%) and/or T1DM (98%). ^‡‡‡^ Significant at the prespecified two-sided α of 0.2. ABC = advanced breast cancer, BC = breast cancer, AI = aromatase inhibitor, CDK = cyclin-dependent kinase, CI = confidence interval, ER = estrogen receptor, ET = endocrine therapy, HER2 = human epidermal growth factor receptor 2, HR = hormone receptor, ITT = intention to treat, MBC = metastatic breast cancer, mTOR = mammalian target of rapamycin, NR = not reported, ORR = overall (complete + partial) response rate, OS = overall survival, PE = primary endpoint, PFS = progression-free survival, PgR = progesterone receptor, pts = patients, T-DM1 = trastuzumab emtansine, TTP = time to progression, and VEGF = vascular endothelial growth factor.

**Table 2:** Phase II and III trials in the neoadjuvant setting reporting outcomes for patients with HR+/HER2+ early-stage breast cancer.

Study Acronym and Phase (Study Population)	No. of pts	Treatment Regimen	Results for pCR ^a^ (% of pts) (95% CI)	Comments
**Neoadjuvant HER2-targeted therapy plus chemotherapy**				
*Trastuzumab vs. lapatinib or trastuzumab + lapatinib*				
NeoALTTO [82] Subgroup HR+/HER2+ (overall HER2+)	80	A: Lapatinib × 6 wks → lapatinib + paclitaxel × 12 wks	pCR: 16.3% (9.0–26.2) *p* = 0.312 vs. B	pCR rate for ER−/HER2+ BC: (A) 33.8% (23.2–45.7) *p* = 0.731 vs. B, (B) 36.5% (25.6–48.5), (C) 61.3% (49.4–72.4); *p* = 0.002 vs. B In the overall population, dual HER2-targeted was better than single HER2-targeted therapy when combined with paclitaxel
	75	B: Trastuzumab × 6 wks → trastuzumab + paclitaxel × 12 wks	pCR: 22.7% (13.8–33.8)	
	77	C: Lapatinib + trastuzumab × 6 wks → lapatinib + trastuzumab + paclitaxel × 12 wks	pCR: 41.6% (30.4–53.4) *p* = 0.013 vs. B	
CHER−LOB [83] Subgroup HR+/HER2+ (overall HER2+)	21	A: Paclitaxel + trastuzumab × 12 wks → fluorouracil + epirubicin + cyclophosphamide + trastuzumab × 4 cycles	Across all treatments pCR: 28.8%	pCR rate for ER−/HER2+ BC across all treatments: 41.3% (no statistical analysis vs. HR+/HER2+ BC) In the overall population, breast and axillary node pCR rates were (A) 25% (13.1–36.9) ^b^, (B) 26.3% (14.5–38.1) ^b^, and (C) 46.7% (34.4–58.9) ^b^; risk ratio of pCR with C vs. A and B: 1.81; *p* = 0.019
	24	B: Paclitaxel + lapatinib × 12 wks → fluorouracil + epirubicin + cyclophosphamide + lapatinib × 4 cycles		
	28	C: Paclitaxel + lapatinib + trastuzumab × 12 wks → fluorouracil + epirubicin + cyclophosphamide + lapatinib + trastuzumab × 4 cycles		
GeparQuinto [84,85] Subgroup HR+/HER2+ (overall HER2+)	170	A: Epirubicin + cyclophosphamide + trastuzumab × 4 cycles → docetaxel + trastuzumab × 4 cycles	pCR: NR 3-year OS: 95.2% (90.1–97.7)	Odds ratio for pCR rate with B vs. A for ER+/HER2+ BC: 0.53 (0.31–0.91) and ER−/HER2+ BC: 0.82 (0.50–1.36) Odds ratio for pCR rate for ER−/HER2+ BC vs. ER+/HER2+ BC: 0.52 (0.35–0.76); *p* = 0.001 In the overall population, pCR rate: (A) 30.3% (25.2–35.8) vs. (B) 22.7% (18.2–27.8); *p* = 0.04 but DFS and OS did not differ significantly between A + trastuzumab × 1 year and B + trastuzumab × 1 year In pts with ER+/HER2+ BC: 3-year OS rate was higher with B + trastuzumab × 1 year than A + trastuzumab × 1 year (hazard ratio OS: 0.32 (0.12–0.87); *p* = 0.019) 3-year OS rate appeared lower in pts with HR−/HER2+ BC than in those with HR+/HER2+ BC in both arms (no statistical analysis)
	171	B: Epirubicin + cyclophosphamide + lapatinib × 4 cycles → docetaxel + lapatinib × 4 cycles	pCR: NR 3-year OS: 97.9% (93.6–99.3)	
CALGB 40601 [44] Subgroup HR+/HER2+ (overall HER2+)	70	A: Paclitaxel + trastuzumab × 16 wks	pCR: 41%	pCR rate for ER−/HER2+ BC: (A) 54%, (B) 79%, (C) 37% Dual HER2-targeted therapy was not significantly different from single HER2-targeted therapy in pts with HR+/HER2+ BC (in pts with HR−/HER2+ BC, pCR was higher with B vs. A; *p* = 0.01) In the overall population, pCR rate: (A) 46, (B) 56%, (C) 32% (A vs. B; *p* = 0.13)
	69	B: Paclitaxel + trastuzumab + lapatinib × 16 wks	pCR: 41%	
	37	C: Paclitaxel + lapatinib × 16 wks	pCR: 29%	
*Trastuzumab/pertuzumab combinations*				
NeoSphere [86] Subgroup HR+/HER2+ (overall HER2+)	50	A: Trastuzumab + docetaxel × 4 cycles	pCR: 20.0% (10.0–33.7)	pCR rate for ER−/HER2+ BC: (A) 36.8% (24.4–50.7), (B) 63.2% (49.3–75.6), (C) 27.3% (16.1–41.0), (D) 30.0% (17.9–44.6) In the overall population, more women given B achieved breast pCR than those given A (*p* = 0.014) or D (*p* = 0.003); C was not as effective as A (*p* = 0.020)
	50	B: Trastuzumab + pertuzumab + docetaxel × 4 cycles	pCR: 26.0% (14.6–40.3)	
	51	C: Trastuzumab + pertuzumab × 4 cycles	pCR: 5.9% (1.2–16.2)	
	46	D: Pertuzumab + docetaxel × 4 cycles	pCR: 17.4% (7.8–31.4)	
TRYPHAENA [87] Subgroup HR+/HER2+ (overall HER2+)	39	A: 5-fluorouracil + epirubicin + cyclophosphamide + trastuzumab + pertuzumab × 3 cycles → docetaxel + trastuzumab + pertuzumab × 3 cycles	pCR: 46.2%	pCR rate for ER−/HER2+ BC: (A) 79.4%, (B) 65.0%, (C) 83.8% pCR rates were higher in patients with HR−/HER2+ BC than in those with HR+/HER2 BC (no statistical analysis) In the overall population, pCR rate: (A) 61.6%, (B) 57.3%, (C) 66.2% (no statistical analysis) The combination of trastuzumab + pertuzumab was generally well-tolerated, with a low incidence of symptomatic left ventricular systolic dysfunction, in all treatment arms
	35	B: 5-fluorouracil + epirubicin + cyclophosphamide × 3 cycles → docetaxel + trastuzumab + pertuzumab × 3 cycles	pCR: 48.6%	
	40	C: Docetaxel + carboplatin + trastuzumab + pertuzumab x 6 cycles	pCR: 50.0%	
**Neoadjuvant ET plus single HER2-targeted therapy**				
ADAPT [88] All pts HR+/HER2+	119	A: T-DM1 × 4 cycles	pCR: 41.0% (*p* < 0.001, A vs. C)	Low pCR with trastuzumab + ET suggests alternative chemotherapy-free regimens are needed T-DM1 had a favorable toxicity profile
	127	B: T-DM1 + ET × 4 cycles	pCR: 41.5% (*p* < 0.001, B vs. C)	
	129	C: Trastuzumab + ET × 4 cycles	pCR: 15.1%	
**Neoadjuvant ET plus dual HER2-targeted therapy**				
*Trastuzumab and lapatinib*				
PAMELA [89] Subgroup HR+/HER2+ (overall HER2+)	77	Trastuzumab + lapatinib + ET × 18 wks (HR+ pts)	pCR: 18%	In HR+/HER2+ BC, the pCR rate was higher in 38 pts with the HER−enriched subtype than in 39 pts with non-HER2-enriched subtypes (32% vs. 5%) pCR rate for ER−/HER2+ BC (no ET): 43%, *p* = 0.0015 vs. HR+/HER2+ BC
TBCRC006 [28] Subgroup ER+/HER2+ (overall HER2+)	39	Trastuzumab + lapatinib + letrozole (ER+ pts)	pCR: 21% pRR: 54%	pCR rate for ER−/HER2+ BC (no ET): 36% pRR rate for ER−/HER2+ BC (no ET): 40% (no statistical analyses)
TBCRC023 [90] Subgroup ER+/HER2+ (overall HER2+)	23	A: Trastuzumab + lapatinib + ET × 12 wks	pCR: 8.7% pRR: 30.4%	pCR rate for ER−/HER2+ BC (no ET): (A) 20.0%, (B) 18.2% pRR rate for ER−/HER2+ BC (no ET): (A) 0%, (B) 18.2% In the overall population, dual HER2-targeted therapy for 24 vs. 12 weeks numerically increased pCR without need for chemotherapy (pCR rate: 27.9% vs. 12.1%)
	39	B: Trastuzumab + lapatinib + ET × 24 wks	pCR: 33.3% pRR: 12.8%	
*Trastuzumab and pertuzumab*				
PerELISA [91] All pts HR+/HER2+	44	Letrozole × 2 wks → letrozole + trastuzumab + pertuzumab (molecular responders ^c^)	pCR: 20.5%	After short-term letrozole: Ki67 reduction (molecular response) identifies pts achieving a meaningful pCR rate without chemotherapy Lack of Ki67 reduction helps identify pts benefiting from chemotherapy + HER2-targeted therapy
	17	Letrozole × 2 wks → paclitaxel + trastuzumab + pertuzumab (molecular nonresponders)	pCR: 81.3% in 16 evaluable	
PHERGain [92] Subgroup HR+/HER2+ (overall HER2+)	NR	A: Docetaxel + carboplatin + trastuzumab + pertuzumab × 6 cycles	NR	pCR rate for ER−/HER2+ BC: B (no ET): 44.3%; *p* = 0.184 vs. HR+/HER2+ BC In the overall population, F-PET identified pts who were more likely to benefit from chemotherapy-free dual HER2-targted therapy: pCR rate: (A) 57.7% (47.4–69.4), (B) (F-PET responders): 37.9% (31.6–44.5), (C) (trastuzumab + pertuzumab ± ET × 2 cycles (F-PET nonresponders) → (A): 25.9% (15.3–39.0)
	NR	B: Trastuzumab + pertuzumab + ET × 6 cycles in F-PET responders after 2 cycles	pCR: 35%	
KRISTINE [93] Subgroup HR+/HER2+ (overall HER2+)	139	A: T-DM1 + pertuzumab	pCR: 35.1%	pCR rate for ER−/HER2+ BC: (A) 54.2%, (B) 73.2% In the overall population, pCR rate for (A) 44.4%, (B) 55.7% (*p* = 0.016)
	138	B: Docetaxel + carboplatin + trastuzumab + pertuzumab	pCR: 43.8%	
**Neoadjuvant ET plus CDK4 and 6 inhibitors plus single or dual HER2-targeted therapy**				
NA-PHER2 [94] All pts HR+/HER2+	30	Trastuzumab + pertuzumab + Palbociclib + fulvestrant	pCR: 27% (12–46) ^d^ Mean Ki67 expression: Baseline: 31.9 (SD 15.7) Surgery: 12.1 (20.0); *p* = 0.013	Mean Ki67 expression was significantly reduced from baseline at week 2 to 4.3 (*p* < 0.0001) and week 16 (time of surgery; *p* = 0.013)
**Neoadjuvant ET plus chemotherapy + dual HER2-targeted therapy**				
NSABP-B52 [95] All pts HR+/HER2+	315 (total)	Docetaxel + carboplatin + trastuzumab + pertuzumab + ET	pCR: 46.1 (*p* = 0.36)	Addition of ET to neoadjuvant therapy nonsignificantly increased pCR without affecting toxicity
		Docetaxel + carboplatin + trastuzumab + pertuzumab	pCR: 40.9	

BC = breast cancer, CDK = cyclin-dependent kinase, CI = confidence interval, DFS = disease-free survival, ER = estrogen receptor, ET = endocrine therapy, F-PET = fluorodeoxyglucose-positron emission tomography, HER2 = human epidermal growth factor receptor 2, HR = hormone receptor, NR = not reported, OS = overall survival, pCR = pathologic complete response, pRR = pathologic response rate (protocol-specified: (ypT0-is + ypT1a-b), pts = patients, SD = standard deviation, T-DM1 = trastuzumab emtansine, and wks = weeks. ^a^ At time of surgery; usually the primary study endpoint, although definitions varied between studies. ^b^ 90% CI. ^c^ Patients with Ki67 relative reduction >20% from the baseline. ^d^ pCR was a secondary endpoint.

**Table 3:** Phase II and III trials in the adjuvant setting, including patients with HR+/HER2+ breast cancer.

Study Acronym (Study Population)	No. of pts	Treatment Regimen	DFS (Primary Endpoint) Hazard Ratio	Comment
**Treatment duration**				
*1 year vs. <1 year*				
Short-HER [98] Subgroup HR+/HER2+ (overall HER2+)	426	Docetaxel + trastuzumab × 3 cycles → 5-fluorouracil + epidoxorubicin + cyclophosphamide + trastuzumab × 3 cycles (9 weeks total)	DFS: 1.15 (90% CI 0.77–1.73)	DFS hazard ratio was similar in patients with HR− BC (1.09 (90% CI 0.67–1.78)) Noninferiority of the shorter regimen not met in total study population (5-year DFS: 85% vs. 88% for long regimen; hazard ratio: 1.13 (90% CI 0.89–1.42)) A shorter trastuzumab administration could be an option for pts who experience cardiac events and for those with a low relapse risk
	201	Anthracycline + cyclophosphamide × 4 cycles → taxane + trastuzumab (1 year total)		
PHARE [99] Subgroup HR+/HER2+ (overall HER2+)	1040	Trastuzumab for 6 months *	ER+: DFS: 1.23 (95% CI 0.92–1.65) PgR+: DFS: 1.24 (95% CI 0.87–1.75)	DFS hazard ratio in patients with ER− BC: 1.34 (95% CI 1.02–1.76) and PgR− BC: 1.28 (95% CI 1.01–1.64) Noninferiority of the shorter regimen not met in total study population (2-year DFS: 94% vs. 91% for long regimen; hazard ratio: 1.28 (95% CI 1.05–1.56))
	1021	Trastuzumab for 1 year *		
SOLD [100] Subgroup ER+/HER2+ (overall HER2+)	711	Docetaxel + trastuzumab × 9 weeks → fluorouracil + epirubicin + cyclophosphamide × 3 cycles	DFS: 1.28 (95% CI 0.96–1.69)	DFS hazard ratio in patients with ER− BC: 1.57 (95% CI 1.14–2.17) Noninferiority of the shorter regimen not met in the total study population (5-year DFS: 88% vs. 91% for long regimen; hazard ratio: 1.39 (90% CI 1.12–1.72)) Cardiac safety better with shorter regimen
	723	Docetaxel + trastuzumab × 9 weeks → fluorouracil + epirubicin + cyclophosphamide × 3 cycles + trastuzumab to 1 year (51 weeks)		
HORG [101] Subgroup HR+/HER2+ (overall HER2+)	165	Epirubicin + 5-fluorouracil + cyclophosphamide × 4 cycles → docetaxel × 4 cycles + trastuzumab × 6 months **	ER+: DFS: 2.20 (95% CI 0.91–5.31) PgR+: DFS: 1.86 (95% CI 0.76–4.55)	DFS hazard ratio in patients with ER− BC: 1.14 (0.48–2.69) and PgR− BC: 1.40 (0.61–3.20) Noninferiority of the shorter regimen not met in the total study population (3-year DFS: 93% vs. 96% for long regimen; hazard ratio: 1.57 (95% CI 0.86–2.10))
	156	Epirubicin + 5-fluorouracil + cyclophosphamide × 4 cycles → docetaxel × 4 cycles + trastuzumab × 1 year **		
PERSEPHONE [102] Subgroup ER+/HER2+ (overall HER2+)	1441	Chemotherapy + trastuzumab × 6 months	DFS: 0.96 (95% CI 0.76–1.20)	DFS hazard ratio in patients with ER− BC: 1.26 (95% CI 0.97–1.64), with no significant effect of ER status Noninferiority of the shorter regimen was met in the total study population (4-year DFS: 89% vs. 90% for long regimen; hazard ratio: 1.07 (95% CI 0.93–1.24); *p* = 0.011) A reduced treatment duration may benefit at least some women with HER2+ BC
	1412	Chemotherapy + trastuzumab × 1 year		
*1 year vs. >1 year*				
HERA [103] Subgroup HR+/HER2+ (overall HER2+)	798	Chemotherapy ± radiotherapy → trastuzumab × 2 years	DFS: 1.05 (95% CI 0.85–1.29)	DFS hazard ratio in patients with HR− BC: 0.93 (95% CI 0.76–1.14) An increased duration of trastuzumab did not improve DFS over a 1-year regimen (DFS hazard ratio: 0.99 (95% CI 0.85–1.14))
	790	Chemotherapy ± radiotherapy → trastuzumab × 1 year		
**Treatment escalation**				
ALTTO [104] Subgroup HR+/HER2+ (overall HER2+)	1203	Lapatinib + trastuzumab ***	DFS vs. trastuzumab: 0.87 (97.5% CI 0.66–1.13)	DFS hazard ratio in patients with HR− BC vs. trastuzumab: 0.82 (95% CI 0.62–1.08) In the total population, combination therapy resulted in a nonsignificant improvement in DFS, which was not clinically meaningful (modest treatment effect + added toxicity)
	1205	Trastuzumab × 12 weeks → lapatinib ***	DFS vs. trastuzumab: 0.92 (97.5% CI 0.71–1.20)	DFS hazard ratio in patients with HR− BC vs. trastuzumab: 1.00 (95% CI 0.77–1.30) In the total population, sequential therapy did not show noninferiority compared with trastuzumab
	1197	Lapatinib ***	Arm discontinued	
	1200	Trastuzumab ***		
APHINITY [105] Subgroup HR+/HER2+ (overall HER2+)	1536	Pertuzumab + trastuzumab + taxane ^†^	DFS: 0.86 (95% CI 0.66–1.13)	DFS hazard ratio in patients with HR− BC: 0.76 (95% CI 0.56–1.04) In the overall population, addition of pertuzumab to chemotherapy and trastuzumab significantly improved DFS (hazard ratio: 0.81 (95% CI 0.67–1.00)) but increased toxicity
	1546	Placebo + trastuzumab + taxane ^†^		
KAITLIN [106] Subgroup HR+/HER2+ (overall HER2+)	516	Anthracycline-based therapy → taxane + trastuzumab + pertuzumab	IDFS: 0.94 (95% CI 0.61–1.44)	IDFS hazard ratio in patients with HR− BC: 1.00 (95% CI 0.66–1.51) In the overall population, addition of pertuzumab to chemotherapy and trastuzumab had no significant effect on IDFS hazard ratio: 0.97 (95% CI 0.72–1.30)
	519	Anthracycline-based therapy → T-DM1 + pertuzumab		
**Other**				
ExteNET [107,108] Subgroup HR+/HER2+ (overall HER2+)	816	Trastuzumab + sequential or concurrent chemotherapy → neratinib × 12 months	2-year IDFS (PE): 0.51 (95% CI 0.33–0.77) 5-year IDFS: 0.60 (95% CI 0.43–0.83)	2-year IDFS (PE) hazard ratio in patients with HR− BC: 0.93 (95% CI 0.60–1.43) 5-year IDFS hazard ratio in patients with HR− BC: 0.95 (95% CI 0.66–1.35) In the total population, neratinib significantly improved 2-year IDFS (PE) when given after chemotherapy and trastuzumab-based adjuvant therapy (IDFS hazard ratio: 0.67 (95% CI 0.50–0.91); *p* = 0.009)
	815	Trastuzumab + sequential or concurrent chemotherapy → placebo × 12 months		
KATHERINE [109] Subgroup HR+/HER2+ (overall HER2+)	540	Neoadjuvant therapy → trastuzumab (+ radiotherapy + hormonal therapy per guidelines)	IDFS: 0.48 (95% CI 0.35–0.67)	IDFS hazard ratio in patients with HR− BC: 0.50 (95% CI 0.33–0.74) The IDFS benefit was consistent irrespective of HR status In the overall population, IDFS was higher with T-DM1 than trastuzumab (IDFS hazard ratio: 0.50 (95% CI 0.39–0.64); *p* < 0.001)
	534	Neoadjuvant therapy → T-DM1 (+ radiotherapy + hormonal therapy per guidelines)		

* Patients were required to have received at least 4 cycles of chemotherapy and had breast-axillary surgery before study entry; additional chemotherapy, hormone therapy, radiation therapy, and treatment schedules were based on clinician choice. ** Patients received hormonal and radiation therapy according to the current standards of care and as decided by the treating clinician. *** Clinicians could administer HER2-targeted therapies at the completion of all chemotherapy or with anthracycline-based chemotherapy preceding the combined administration of HER2-targeted therapies plus taxane (paclitaxel or docetaxel) or (in North America) combined with an anthracycline-free regimen (docetaxel plus carboplatin × 6 cycles). ^†^ HER2-targeted therapy was given in the following regimens: 5-fluorouracil + anthracycline + cyclophosphamide → taxane + HER2-targeted therapy or cyclophosphamide + anthracycline → taxane + HER2-targeted therapy or docetaxel plus carboplatin + HER2-targeted therapy; patients with HR+ BC received standard endocrine therapy starting at the end of chemotherapy; radiotherapy was given as clinically indicated at the end of chemotherapy and concomitantly with HER2-targeted therapy. BC = breast cancer, CI = confidence interval, DFS = disease-free survival, ER = estrogen receptor, HER2 = human epidermal growth factor receptor 2, HR = hormone receptor, IDFS = invasive disease-free survival, PE = primary endpoint, PgR = progesterone receptor, pts = patients, and T-DM1 = trastuzumab emtansine.
